# Profiling of low molecular weight proteins in plasma from locally irradiated individuals

**DOI:** 10.1093/jrr/rru007

**Published:** 2014-02-24

**Authors:** Reetta Nylund, Elina Lemola, Sonja Hartwig, Stefan Lehr, Anna Acheva, Jutta Jahns, Guido Hildebrandt, Carita Lindholm

**Affiliations:** 1 STUK – Radiation and Nuclear Safety Authority, Laippatie 4, PO Box 14, 00881 Helsinki, Finland; 2 Institute for Clinical Biochemistry and Pathobiochemistry, German Diabetes Center, Auf'm Hennekamp 65, 40225 Düsseldorf, Germany; 3 Department of Radiotherapy and Radiation Oncology, University of Leipzig, Stephanstrasse 9a, 04103 Leipzig, Germany; 4 Department of Radiotherapy, University of Rostock, Suedring 75, 18059 Rostock, Germany

**Keywords:** ionizing radiation, radiation proteomics, clastogenic factors, low molecular weight plasma proteins, two-dimensional difference gel electrophoresis (2DE-DIGE)

## Abstract

In studies reported in the 1960s and since, blood plasma from radiation-exposed individuals has been shown to induce chromosome damage when transferred into lymphocyte cultures of non-irradiated persons. This effect has been described to occur via clastogenic factors, whose nature is still mostly unknown. We have previously examined clastogenic factors from irradiated individuals by looking at plasma-induced DNA damage in reporter cells. Plasma was tested from ca. 30 locally exposed clinical patients receiving fractionated radiation treatment, as well as from three radiological accident victims exposed in 1994, albeit sampled 14 years post-accident. In the current work, proteome changes in the plasma from all subjects were examined with 2D gel electrophoresis-based proteomics techniques, in order to evaluate the level of protein expression with respect to the findings of a clastogenic factor effect. No differences were observed in protein expression due to local radiation exposure (pre- vs post-exposure). In contrast, plasma from the radiation accident victims showed alterations in the expression of 18 protein spots (in comparison with plasma from the control group). Among these, proteins such as haptoglobin, serotransferrin/transferrin, fibrinogen and ubiquitin-60S ribosomal protein L40 were observed, none of them likely to be clastogenic factors. In conclusion, the proteomics techniques applied were unable to identify changes in the proteome of the locally irradiated patients, whereas such differences were observed for the accident victims. However, association with the clastogenic effect or any specific clastogenic factor remains unresolved and thus further studies with more sensitive techniques are warranted.

## INTRODUCTION

In studies dealing with the biological effects of ionizing radiation, an interest in the molecular contents of the blood plasma emerged in the 1960s. Plasma from irradiated individuals was shown to induce chromosomal aberrations when cultured with cells from an unexposed person. The term ‘clastogenic plasma factors’ was generated for this phenomenon. Clastogenic factors (CFs) were initially observed in plasma obtained from therapeutically or accidentally irradiated individuals [1–3]. Later, similar findings were obtained, among others, in studies of A-bomb survivors, Chernobyl cleanup workers, and children exposed to radiation from the Chernobyl accident [4–6]. It also became evident that there is a large individual variation in CF formation. Studies of CFs originate from observations on the plasma from irradiated persons, but the phenomenon is, however, common in a large number of health defects (reviewed in [7]).

The earlier CF studies revealed that the size of the clastogenic plasma factors should focus on low molecular weight (LMW) molecules [7]. These studies suggested also that CFs are not composed of only one type of molecule, but that many different substances, such as lipid peroxidation products, nucleotides of inosine, and cytokines, were involved [7]. It was proposed that CF formation is a self-sustaining process in which superoxide acts as the initiator in the formation of CFs, which in turn are capable of generating superoxide. This mechanism would explain the persistence of CF in the plasma of persons exposed to ionizing radiation years or decades ago. CFs belong to non-targeted effects, i.e. secreted factors produced in irradiated cells that are capable of inducing effects in non-irradiated cells [8].

We have previously performed a CF study on locally exposed individuals with benign conditions, before and after they had received ionizing radiation to small treatment volumes [9]. Plasma-induced markers that represent DNA damage, i.e. chromosomal aberrations, micronuclei and phosphorylated H2AX protein (γ-H2AX), were investigated in reporter cells. Local radiation exposure had seemingly no effect on inducing such markers, whereas an increase in the yield of chromosomal aberrations in reporter cells was induced by plasma from two radiological accident victims, with plasma samples taken more than ten years post-exposure. In the present paper, the very same plasma samples from locally irradiated persons were analyzed with 2D gel electrophoresis (2-DE)-based proteomics methods in order to assess changes in the expression level of plasma proteins and to evaluate the findings with respect to knowledge regarding CFs.

## MATERIALS AND METHODS

### Plasma samples from locally exposed individuals, controls, and radiation accident victims

The human subjects in this study and blood sampling techniques have been earlier described by Lindholm *et al*. [9].The authorizing body for the human experiments was the University of Leipzig. Blood sampling was performed according to the Declaration of Helsinki. Briefly, from November 2007–November 2008, blood samples were obtained at the University Hospital Leipzig, Germany, from clinical patients who had received exposures to fractionated radiation treatment with very small or small treatment volumes/areas and/or using low single and total radiation doses. Informed consent was obtained from patients along with an ethics statement from the local ethics board prior to blood sampling. Three patient groups were examined, each composed of between 6 and 12 people. Group I (6 people) consisted of patients with marginal resected basal cell carcinoma treated with adjuvant^192^Iridium high dose-rate plesio-brachytherapy using Leipzig applicators (diameter 15–30 mm). The radiation dose was 5.0 Gy per fraction (two times per week) until a total dose of 40.0 Gy was reached in 4 weeks. Treatment volumes ranged from 1.0–3.5 cm^3^. Group II patients (12 people) suffered from painful osteoarthritis of the knee and were treated with radiotherapy with high-energy photons (6 MV) using parallel-opposed treatment fields. Six fractions (two times per week) with single doses of 1.0 Gy were applied, leading to a total dose of 6.0 Gy over 3 weeks. Treatment volumes ranged from 800–1150 cm^3^. Group III patients (11 persons) suffered from painful tendinitis of either the elbow (epicondylopathia humeri) or the heel (calcaneal spur) and received orthovoltage therapy (120–150 kV). Six fractions (two times per week) with single doses of 1.0 Gy were applied, leading to a total dose of 6.0 Gy over 3 weeks. Treatment volumes ranged from 80–160 cm^3^.

Additionally, Group IV (controls) consisted of 10 healthy donors from Leipzig. Two different samples were obtained from each donor at 3-week intervals to identify differences in sampling times and to assess control level variation in assays.

Information on age, sex, smoking, duration of the symptoms, previous radiotherapy, and additional steroid and non-steroidal anti-inflammatory drug (NSAID) treatment was collected from each person (Table 1, modified from [9]). Blood sampling was carried out immediately before the first and directly after the last dose fraction was delivered i.e. 3–4 weeks after the beginning of the treatment. Each blood sample consisted of 20–30 ml Li-heparin blood. Plasma was extracted from each sample (centrifugation of the blood 15 min at 3000 rpm) and stored in 1.5 ml aliquots at − 80°C. Frozen plasma samples were shipped from Leipzig to STUK, Finland by a courier.

**Table 1.** Background information on locally exposed individuals (Groups I–III), controls and radiation accident victims (modified from [9]).

	Number of persons	Mean age (range) in years	Males/females	Smokers (non-/NA/current /ex)^a^	Steroid treatment (no/NA/yes)^a^	Non-steroidal anti-inflammatory drug (no/NA/yes)^a^	Mean duration of symptoms (range) in months^b^
Group I	6	71 (65–78)	3/3	4/1/0/1	5/1/0	6/0/0	11 (1–24)
Group II	12	68 (42–85)	5/7	5/1/1/5	5/1/6	2/2/8	62 (12–150)
Group III	11	56 (40–73)	5/6	4/0/1/6	7/3/1	4/5/2	9 (2–18)
Controls	10	42 (20–59)	5/5	6/0/4/0			
Radiation accident victims	3	38 (27–48)	2/1	Estimated protracted dose [10]: 2.7; 0.5; 1.0 Gy			

NA = Data not available. ^a^No. of persons. ^b^Data based on all patients except one Group I patient for whom data are missing.

Furthermore, plasma from three victims of the 1994 radiation accident in Kiisa, Estonia [10] was collected in 2008. The victims were exposed to ionizing radiation from a stolen ^137^Cs source, Persons #1 and #2 with protracted exposure over 4 weeks and #3 with non-homogeneous exposure for few hours. The estimated whole-body dose was 2.7 Gy for Person #1 and 0.5 Gy for Person #2, and the partial-body dose for Person #3 was estimated to be 1.0 Gy. Plasma samples were collected as described above.

### Optimization of sample preparation for gel electrophoresis

**Fig. 1.**
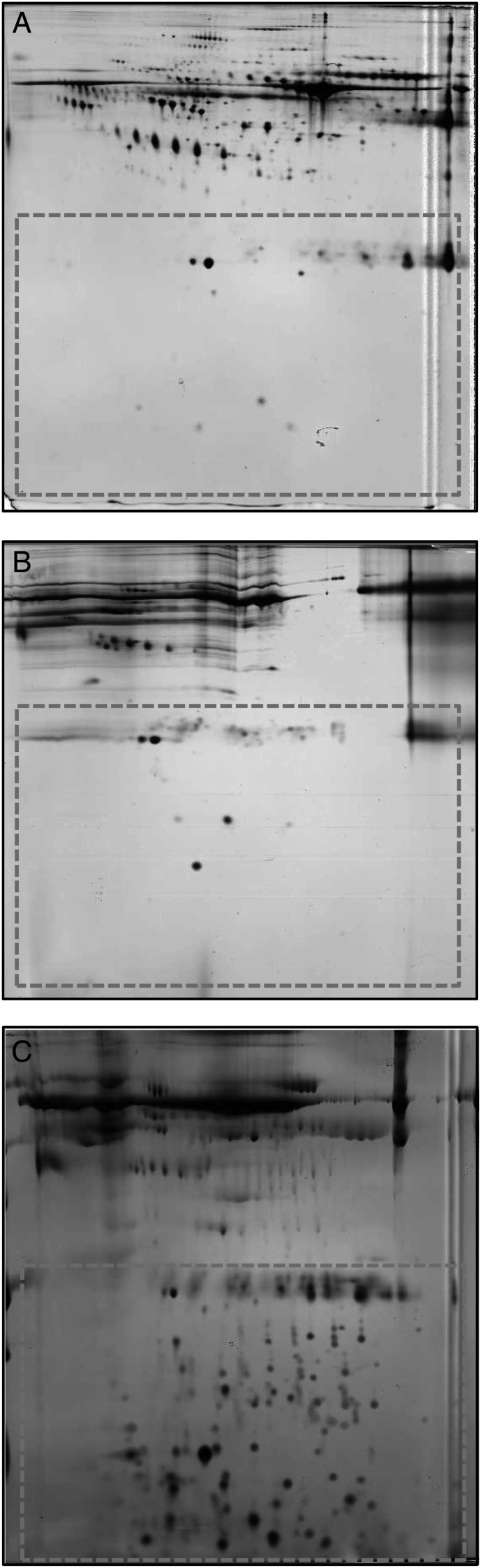
Two-dimensional gels from human plasma samples. (**A**) Native human plasma (total protein 50 µg, pH 4–7, 12.5% SDS-PAGE). (**B**) Human plasma filtered with 30-kDa cut-off filters (total protein 50 µg, pH 3–10, 15% SDS-PAGE). (**C**) Human plasma treated with 20% ACN, 25 mM NH_4_HCO_3_ and filtered with 30 kDa and 3 kDa cut-off filters (total protein 22 µg, pH 3–10, 15% SDS-PAGE).

The large majority of plasma consists of a few known proteins [11], such as albumin and immunoglobulins and, thus, only a few LMW proteins are detected in a 2-DE map of pure plasma, as was prepared in this study (Fig. 1A). Previous studies of CFs have used either 10-kDa or 30-kDa cut-off filters to remove high molecular weight plasma compounds, possibly interfering with the cell culture. However, only a few proteins were detected in the 2-DE map of plain human plasma filtered with 30-kDa cut-off filters (Fig. 1B) and, thus, further sample enrichment was required. Different buffers, treatment conditions, sample-to-buffer concentrations, and cut-off filters were tested so as to be able to produce optimal 2-DE gels. The modified system of Tirumalai *et al*. [12] produced the best resolution of LMW proteins in the 2-DE maps (Fig. 1C, area surrounded with dashes) and was selected as the treatment protocol for study samples to investigate proteome changes potentially associated with the effect of CFs. The limited quantity of plasma sample required and the highest gain of LMW spots in the 2-DE map were the main arguments supporting the selection of this approach.

Individual plasma samples for proteomics analysis were treated with the modified protocol by Tirumalai [12]. Briefly, 2 ml of plasma was diluted and mixed with 12 ml of 20% acetonitrile (ACN) and 25 mM ammonium bicarbonate (NH_4_HCO_3_) for 2 h with occasional vortexing and sonication. The mixture was filtered through 30-kDa Amicon Ultra-15 centrifugal filters (Millipore, USA) at 3360 × *g* until > 95% of the input plasma–buffer mixture had passed the membrane. The filtrate was concentrated and desalted by filtering with 3-kDa Amicon Ultra-15 centrifugal filters (Millipore) until the volume of the remaining liquid was ca. 150 µl. The treatment was performed for a 2 ml plasma volume for each sample: two timepoints for patients and controls, one for accident victims. In total, 81 plasma samples were prepared and analyzed.

### 2D difference gel electrophoresis

Protein concentrations were measured using the Bradford method, and the sample pH was adjusted to 8.5 with NaOH to enable Cy-labeling. From each treated sample, 25 µg of total protein was used for analysis. An internal standard was prepared by pooling a portion of all samples. Samples were labeled with Cy-dyes using minimal dye labeling protocol according to manufacturer's instructions (GE Healthcare, USA), i.e. 200 pmol of dye was used for 25 µg protein, and labeling was performed for 30 min on ice in the darkness and quenched with 10 mM lysine for 10 min in the darkness. The internal standard was always labeled with Cy2-dye and a dye-swap protocol was used for pre- and post-irradiation samples (i.e. Person 1: before irradiation Cy3, after irradiation Cy5; Person 2 vice versa etc.). Pre- and post-irradiation samples from the same study subject were pooled together with the internal standard and separated in the same gel. Plasma samples from the radiation accident victims were labeled either with Cy3- or Cy5-dye and separated with the internal standard in a 2-DE gel.

Samples were mixed with rehydration buffer (9 M Urea, 2% Chaps, 0.5% IPG buffer pH 3–10 NL, 65 mM dithioreitol (DTT), and a trace of bromophenol blue) and incubated for 30 min at room temperature. The isoelectric focusing was performed using an IPGphor 3 apparatus (GE Healthcare) and 24-cm IEF strips pH 3–10 NL (GE Healthcare)at up to 95 kVhrs as described earlier [13]. SDS-PAGE was performed overnight (ca. 17 h) at 20°C using 15% gels in an Ettan DALTsix Electrophoresis system (GE Healthcare) with the following settings: the first 2 h at 10 mA/1 W/gel and the remaining time at 15 mA/2 W/gel.

After electrophoresis the gels were scanned with Typhoon Trio scanner (GE Healthcare) with the appropriate excitation and emission wavelengths for Cy2-, Cy3-, and Cy5-dyes. The PMT voltages were optimized in such a manner that the maximum signal intensity was within the 15% range for all of the dyes.

### Data analysis

The acquired datasets were cropped with ImageQuant Tool-software (GE Healthcare) to contain approximately the same pattern of proteins in all cases. Although cut-off filters of 30 kDa were used, a part of the plasma albumin (ca. 67 kDa) was also recovered in the ultrafiltrate, and thus the area of analysis in the 2-DE map was limited to molecules below ca. 50 kDa. The datasets were imported to DeCyder 7.0 software (GE Healthcare), in which the batch processor was used to detect the spots and match them against a selected master gel. The number of spots was estimated to 10 000, and a spot volume intensity of 5000 was used as a cut-off filter. Matched spots were quickly checked manually in the DeCyder Biological Variation Analysis module (BVA), and the workspace was imported to the DeCyder Extended Data Analysis module (EDA) for statistical analysis. The protein spots found in at least 20% of spot maps were included in the analysis (ca. 2600 spots). The Student's t-test (with and without false discovery rate (FDR) correction) was used to find differentially expressed protein spots. Principal component analysis (PCA) was also performed for the spot maps. The lists containing spots demonstrating statistically significant differences were imported back to the BVA. The results were evaluated numerically based on the average ratio between the sample group mean values for each protein spot as well as visually for identification of possible background artifacts.

Data obtained from the DeCyder were analyzed with respect to the available information on study subjects. First, patient data were analyzed based on local irradiation treatment groups by comparing the pre- and post-irradiation samples. Second, as previous investigations of the samples indicated that individual steroid treatment influenced the clastogenic effect [9], the data were also analyzed with respect to earlier steroid treatment. Patient material was divided into a steroid-treated group consisting of 7 patients (in total 14 gels, pre- and post-irradiation) and a non-treated group of 22 patients (44 gels in total, respectively). A third comparison was performed between radiation accident victims (three persons, three gels) and the healthy controls (10 persons, in total 20 gels).

### Protein identification using mass spectrometry

Gel plugs were picked manually from several silver-stained gels and destained (30 mM potassium ferricyanide, 100 mM sodium thiosulfate) at STUK, Finland, after which gel plugs were shipped to the German Diabetes Center, Germany for protein identification. For in-gel digestion, gel pieces were washed for 10 min in 10 mM ammonium biocarbonate (NH_4_HCO_3_) buffer and buffer containing 50% acetonitrile (ACN) (1:1, v/v). Neat ACN was added and removed to dehydrate the gel pieces. The dry gel pieces were rehydrated in an ice-cold solution of 3.5 ng/µl trypsin (sequencing grade, Promega, USA) in 10 mM NH_4_HCO_3_ for 30 min. Proteins were digested at 37°C for 4 h. Peptides were extracted for 30 min with 10 µl of 0.1% trifluoroacetic acid (TFA) and directly applied to a MALDI Pre-spotted AnchorChip target (Bruker Daltonics, Germany) according to the manufacturer's instructions.

A Time-of-flight Ultraflex-ToF/ToF mass spectrometer (MS) (Bruker Daltonics) was used to analyze samples. Acquired mass spectra were automatically calibrated and annotated using Compass 1.3 software (Bruker Daltonics) and .xml formatted peak lists were transferred to Proteinscape 2.1 (Bruker Daltonics). MS peptide mass fingerprint and fragment spectra from each individual spot were combined and used to search a human subset of the Swiss-Prot (Sprot_2011; 532 146 sequences; 188 719 038 residues; 20 249 human sequences) non-redundant database using the Mascot search engine (Version 2.2, Matrix Science Ltd, London, UK) in consideration of the following settings: enzyme ‘trypsin’, species ‘human’, fixed modifications ‘carbamidomethyl’, optional modifications ‘methionine oxidation’ and missed cleavages ‘1’. Mass tolerance was set to 50 ppm for peptide and 0.7 Da for fragment spectra. Using these settings, a combined mascot score of > 70 was taken as significant (*P* < 0.01). Calculated pI and molecular mass data were obtained by Mascot. For peptides matching to different protein species, we used the following reporting criteria: The experimental pI and molecular mass taken from the 2D-gels were compared with the theoretical data of the protein species. If no conflicts in molecular mass or pI were found, the protein species with the highest mascot score was reported.

## RESULTS

### Analysis of plasma from locally irradiated subjects

**Fig. 2.**
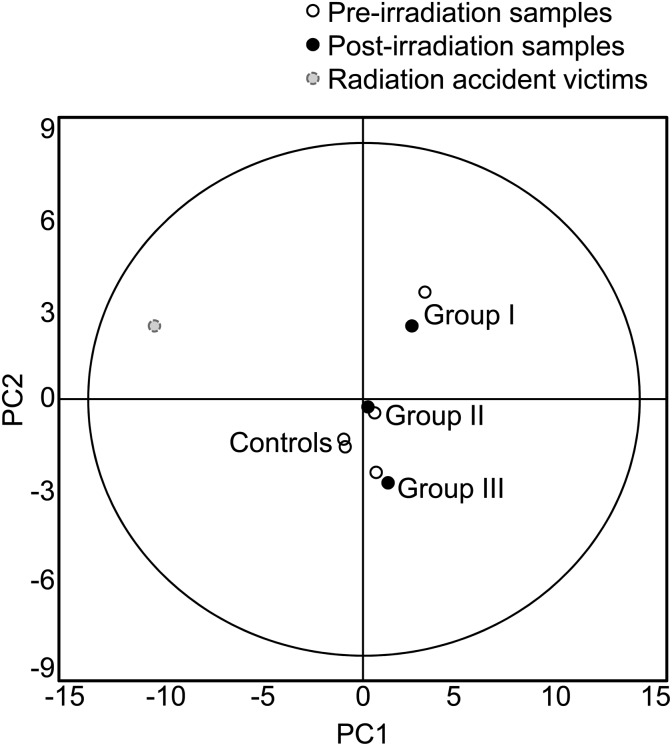
PCA of different experimental groups. No differences were observed due to local exposure of patients (Groups I, II and III) or controls (samples were obtained 3–4 weeks apart), while radiation accident victims clearly differed from other individuals.

Statistical testing using a paired Student's t-test was performed on protein expression profiles of pre- and post-irradiation samples obtained from the locally irradiated individuals in each study group. No differences between the two sets were observed. Furthermore, there were no marked discrepancies in the PCA between the pre- and post-samples in any of the experimental patient groups (Fig. 2).

**Fig. 3.**
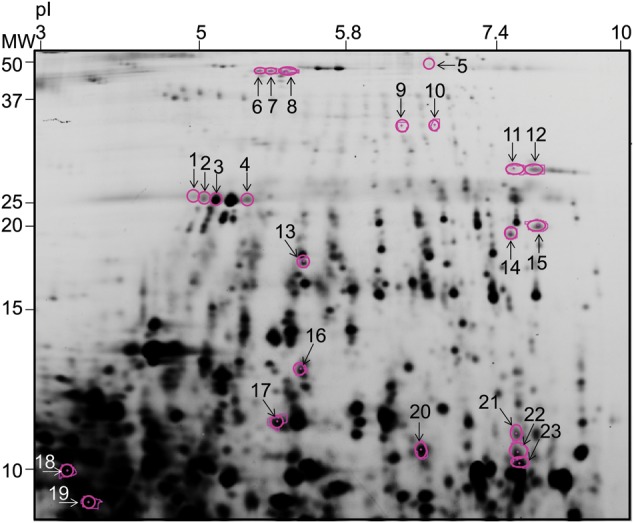
A plasma 2-DE-gel was cut below ca. 55 kDa for analysis. Numbered spots (1–5) represent proteins spots that displayed alterations in steroid-treated individuals in comparison with non-steroid-treated individuals. Numbered spots (6–23) represent proteins spots that displayed alterations in radiation accident victims in comparison with control group (IV).

Plasma samples were also analyzed based on earlier individual steroid treatment. Patients with reported steroid treatment were pooled to the same group, while the non-steroid treated patients served as controls. The expression of five proteins was shown to diverge between (t-test without FDR correction, *P* < 0.01 and a minimum of 1.5-fold ratio difference in protein abundance) The location of these protein spots (no. 1–5) in the 2-DE gel are presented in Fig. 3. The expression of all spots was higher in steroid-treated individuals than in non-treated individuals or in the control group. Three of these proteins spots were identified as apolipoprotein A-1, which is a major component of plasma high-density lipoprotein (HDL) and participates in the reverse transport of cholesterol from tissues to the liver. Two spots remained unidentified. Details of the protein identifications as well as fold ratios between the groups are presented in Table 2.

**Table 2.** Spots having an altered expression level (*P* < 0.01, no FDR correction) in steroid-treated individuals in comparison with non-steroid-treated individuals

Spot^a^	Protein identification	Accession^b^	Mascot score	Fold change^c^	*P*-value^d^
1	Apolipoprotein A-1	P02647	86	1.54	0.003
2				1.77	0.002
3	Apolipoprotein A-1	P02647	278	1.51	0.008
4	Chain A, Crystal Structure of Lipid-Free Human Apolipoprotein	gi|90 108664	87	1.62	0.004
5				1.67	0.002

Expression of all proteins was higher in steroid-treated persons than in non-steroid-treated persons. ^a^Spot number as displayed in Fig. 3. ^b^Database accession code (UniProt or NCBI ID). ^c^Fold ratios are shown as a ratio of treated/non-treated. ^d^t-test *P*-values (no FDR correction).

### Analysis of plasma from radiation accident victims

Plasma from the radiation accident victims was compared with plasma from the control group (Group IV). Expression of 18 proteins (t-test with FDR correction, *P* < 0.05 and a minimum of 1.5-fold ratio between protein abundance mean values of the groups) showed alterations when compared with the plasma from the control group. The location of these protein spots (no. 6–23) in the 2-DE gel are presented in Fig. 3. Details of protein identifications, fold ratios and *P*-values of the analyses are presented in Table 3. Among the downregulated molecules, haptoglobin (no. 16) and serotransferrin/transferrin (no. 8 and 12/no. 11, respectively) were distinguished, while ubiquitin-60S ribosomal protein L40 was found to be both down- and upregulated (no. 19 and 23, respectively). Seven spots (no. 9, 10, 13, 15, 17, 20 and 22) were identified as fibrinogen alpha chain/fibrin alpha C-term fragments displaying both down- and upregulated expression, while five protein spots remained unidentified due to the limited amount of material (no. 6, 7, 14, 18 and 21). The proteins affected in the plasma ofthe three radiation accident victims are common plasma molecules: haptoglobin is expressed by the liver and secreted into plasma, where its primary function is to combine with free plasma hemoglobin to inhibit its oxidative activity and prevent loss of iron through the kidneys. A recent study reported that haptoglobin also prevents against hemoglobin-caused stress and, thus, downregulation of haptoglobin could allow hemoglobin-induced oxidative stress [14]. Serotransferrin/transferrin is also expressed by the liver and secreted into the plasma. It functions as an iron-binding transport protein and it is responsible for the transport of iron from sites of absorption and heme degradation to storage and utilization. Fibrinogen alpha chain is a plasma protein functioning in yielding monomers, which polymerize into fibrin, and act as a cofactor in platelet aggregation. Ubiquitin-60S ribosomal protein L40 is a component of the 60S subunit of the ribosome functioning in protein synthesis.

**Table 3.** Spots having an altered expression level (*P* < 0.05, with FDR correction) in radiation accident victims in comparison with controls

Spot^a^	Protein identification	Accession^b^	Mascot score	Fold change^c,d^	*P*-value^e^
6				−2.90	0.016
7				−2.88	0.023
8	Serotransferrin	P02787	107	−2.78	0.007
9	Fibrinogen Alpha Chain	P02671	199	−1.65	0.033
10	Fibrinogen Alpha Chain	P02671	143	−1.64	0.025
11	Transferrin	gi|33 9486	89	−2.36	0.027
12	Serotransferrin	P02787	116	−2.83	0.024
13	Fibrinogen Alpha Chain	P02671	253	−1.50	0.039
14				−4.61	0.007
15	Fibrinogen Alpha Chain	P02671	90	−2.13	0.040
16	Haptoglobin	P00738	154	−2.50	0.045
17	Fibrin Alpha C Term Fragment	gi|22 3057	109	−1.76	0.047
18				−2.52	0.023
19	Ubiquitin-60S ribosomal protein L40	P62987	202	−4.37	0.047
20	Fibrinogen Alpha Chain	P02671	164	1.75	0.007
21				2.04	0.007
22	Fibrinogen Alpha Chain	P02671	128	2.80	0.007
23	Ubiquitin-60S ribosomal protein L40	P62987	224	1.72	0.041

^a^Spot number as displayed in Fig. 3. ^b^Database accession code (UniProt or NCBI ID). ^c^Fold ratios are shown as a ratio of radiation accident victim/control. ^d^‘ − ’ sign expresses downregulation in radiation accident victims in comparison with the control group. ^e^t-test *P*-values (with FDR correction).

Differences in the plasma proteome of the radiation accident victims in comparison with other individuals can also be detected from the PCA (Fig. 2). The PCA of individual spot maps indicated that especially one of the radiation accident victims differed from the other persons (data not shown). The person in question had received heterogeneous partial body dose of ∼1 Gy during a few hours.

## DISCUSSION

In the current study, the 2-DE-based plasma analysis of the locally irradiated individuals revealed no differences between the pre- and post-treatment proteomes. This result is in agreement with the earlier chromosomal aberrations experiments, where CF-effect was not induced with patient plasma [9]. There may be several reasons to explain the outcome: first, the sensitivity of the detection technique may not be sufficient to distinguish low abundance molecules. Second, the number of investigated locally exposed persons was rather low and, thus, the individual variation may have influenced the detection of molecules of interest. Third, the irradiated tissue volumes remained relatively small in all locally irradiated groups and, thus, only a limited amount of potential irradiation responders (e.g. cells in the blood circulation or tissue) were exposed. Furthermore, confounders such as steroid treatment may have prevented identification of effects from the irradiation. Finally, the selected timepoint of post-irradiation blood sampling (immediately after the last fraction) may not have been optimal considering the time required for the induction of CFs or other differences in the plasma proteome. In contrast to patient results, plasma from the radiation accident victims presented clear changes in protein expression when compared with plasma from control persons, corroborating reports of long-term effects of radiation exposure on plasma [7]. These findings were also congruent with our earlier report of the clastogenic characteristics of the accident victim plasma, i.e. ability to induce chromosomal aberrations *in vitro* [9]. Although changes in the proteome were observed in the accident victim plasma, the association to the clastogenic effect or any specific CF remained unresolved.

The detection of radiation-induced biomarkers using the proteomics approach for human plasma or serum has been applied in only a few studies involving radiotherapy of cancer patients [15–17]. In all these studies, none of the identified proteins was similar to those found in our patient material. The main differences found in the above studies in relation to our work lie in the patient material, as well as varying, but typically higher, doses and dose volumes. Furthermore, the time interval between exposure and sample collection was shorter in the present study than in the other reports, suggesting that potential effects might be more easily detectable after a longer interval. For instance, Widlak *et al*. [17] found changes in serum profiles 4–6 weeks after the radiotherapy was ceased, whereas no differences were observed two weeks after the beginning of the treatment. A longer interval is also suggested from current knowledge of CFs, which are typically still found years after the exposure e.g. among A-bomb survivors and persons exposed in the Chernobyl accident.

Several of the molecules observed in the long-term samples from the radiation accident victims have been reported in animal and *in vitro* study set-ups, although with differential regulation, i.e. both up- and downregulation as a function of time after exposure. In the present study, haptoglobin and serotransferrin/transferrin were downregulated, while expression of fibrinogen was both up- and downregulated depending on the spot. Magic *et al*. [18] reported an acute increase in haptoglobin and fibrinogen expression in rat plasma after irradiation to a total body dose of 4–12 Gy. This increase was observed a few days after the exposure, while later timepoints were not examined. Kim *et al*. [19] reported varying effects in transferrin receptors depending of the time after the single dose exposure to the 5 Gy using an *in vitro* model. Chen *et al*. [20] examined mice bone marrow after *in vivo* whole-body exposure to 4 Gy using 2-DE-based proteomics and found serotransferrin, haptoglobin and apolipoprotein A-1 to be upregulated 24 h after the exposure. Britten *et al*. [21] exposed rats to a 2-Gy dose of ^56^Fe radiation and reported 10 serum biomarkers, one of which was haptoglobin downregulation due to radiation exposure. Guipaud *et al*. [22] exposed mice to a 40-Gy dose, collected samples at several time points for up to one month and examined serum proteome changes using 2DE-DIGE-based proteomics) and reported several isoforms of haptoglobin to be upregulated. The same research group has also later reported upregulation of haptoglobin [23] as well as post-translational modifications in certain mouse serum proteins [24] after exposure to ionizing radiation. Donnadieu-Claraz *et al*. [25] exposed pigs to a total body dose of 2–6 Gy and found variations in blood iron levels after the exposure, depending on the timepoint. The preceding examples demonstrate that there is no clear-cut effect in protein expression after radiation exposure, as it is dependent on the type of irradiation, post-exposure time and other circumstances. In the present study, the radiation victims were exposed several years ago, and thus direct comparison with the illustrated reports is not feasible. It is evident that the expression profiles of the radiation victims diverge clearly from the control group. However, they do not provide direct evidence for molecules possessing long-term clastogenic activity. The plasma proteomic profile can rather be considered as a reflection of oxidative stress that has occurred in these subjects due to the exposure.

Biochemical analyses have revealed that the radiation-induced CF is not a single factor, but instead a mixture of pro-oxidant substances that damage chromosomes [7]. Assuming that an increase in the expression level would be an indicator of CFs, the results of the present study suggest that no such factors were identified in the plasma proteome. Instead, the affected proteins appear to be universal responders to irradiation in multiple systems, suggested in this study to be affected also in humans. Since chromosomal aberrations were observed in the earlier study [9], future studies with more sensitive proteomics techniques than used in this study are warranted. Further investigation of the etiology of the plasma CFs should also focus on other molecules than proteins using sensitive and specific tests. Finally, these studies would benefit from larger numbers of radiation accident victims and/or patients with larger exposure volumes.

## FUNDING

This work was supported by the NOTE IP 036465 (FI6R), the Euratom Specific Programme for Research and Training on Nuclear Energy, the 6th Framework Programme of the European Commission, STUK – Radiation and Nuclear Safety Authority, the German Diabetes Center, the University of Leipzig and the University of Rostock.
